# Tabulate Corals after the Frasnian/Famennian Crisis: A Unique Fauna from the Holy Cross Mountains, Poland

**DOI:** 10.1371/journal.pone.0149767

**Published:** 2016-03-23

**Authors:** Mikołaj K. Zapalski, Błażej Berkowski, Tomasz Wrzołek

**Affiliations:** 1 University of Warsaw, Faculty of Geology, Warsaw, Poland; 2 Adam Mickiewicz University, Institute of Geology, Poznań, Poland; 3 University of Silesia, Faculty of Earth Sciences, Sosnowiec, Poland; Institute of Vertebrate Paleontology and Paleoanthropology Chinese Academy of Sciences, CHINA

## Abstract

Famennian tabulate corals were very rare worldwide, and their biodiversity was relatively low. Here we report a unique tabulate fauna from the mid- and late Famennian of the western part of the Holy Cross Mountains (Kowala and Ostrówka), Poland. We describe eight species (four of them new, namely ?*Michelinia vinni* sp. nov., *Thamnoptychia mistiaeni* sp. nov., *Syringopora kowalensis* sp. nov. and *Syringopora hilarowiczi* sp. nov.); the whole fauna consists of ten species (two others described in previous papers). These corals form two assemblages—the lower, mid-Famennian with *Thamnoptychia* and the upper, late Famennian with representatives of genera ?*Michelinia*, *Favosites*, *Syringopora* and ?*Yavorskia*. The Famennian tabulates from Kowala represent the richest Famennian assemblage appearing after the F/F crisis (these faunas appear some 10 Ma after the extinction event). Corals described here most probably inhabited deeper water settings, near the limit between euphotic and disphotic zones or slightly above. At generic level, these faunas show similarities to other Devonian and Carboniferous faunas, which might suggest their ancestry to at least several Carboniferous lineages. Tabulate faunas described here represent new recruits (the basin of the Holy Cross mountains was not a refuge during the F/F crisis) and have no direct evolutionary linkage to Frasnian faunas from Kowala. The colonization of the seafloor took place in two separate steps: first was monospecific assemblage of *Thamnoptychia*, and later came the diversified *Favosites-Syringopora-Michelinia* fauna.

## Introduction

Middle and Late Devonian (Frasnian) reefal structures were distributed on a global scale. Most of them were dominated by microbialites, calcified cyanobacteria, stromatoporoids and tabulate corals [1, 2]. Tabulates represent a group which was markedly diversified during this time. The number of species was relatively high, and in many areas exceed 50 (Holy Cross Mountains: [3], Kuznetsk Basin: [4], Russian Platform and W slopes of Ural Mts.: [5]) or even 80 (Ardennes: [6, 7]) for Givetian and Frasnian counted together.

The Frasnian/Famennian crisis was marked by a significant collapse of reefal environments [1, 8]. Famennian reefal structures described up to date are rare, and mostly are formed by non-skeletal, mainly microbial communities [1, 2]. Tabulate corals are one of the groups that severely declined after the F/F event, and as a result Famennian tabulates are scarce [9]. Similarly to colonial rugose corals [10–12] tabulates slowly recovered in the late Famennian ("Strunian") and subsequently in early Carboniferous [1, 13, 14], but their diversity never again reached previous levels. Famennian tabulate faunas overall have very low biodiversity—only two species are known so far from the Holy Cross Mountains [9, 15], also two from Omolon Region (NE Russia; [16]), or four species known from the Ardennes [6].

The aim of this paper is to report further discoveries of upper Famennian tabulate corals from the Holy Cross Mountains in Central Poland, based on large collections of tabulates and rugosans acquired over the years by the University of Silesia. We have identified a total of 10 species (eight in this paper) and this is so far the most diversified Famennian tabulate fauna ever described. This study also completes the recent studies on taxonomy of Devonian tabulates from the Southern Region of the Holy Cross Mountains [3, 9, 17].

## Material and Methods

The specimens were collected as whole colonies, or colony fragments; of 14 more or less complete colonies and 31 fragments of branches 55 thin sections and 14 acetate peels were prepared. No permits were required to collect these specimens. Thin sections and peels were observed in transmitted light and in the dark field under stereoscopic microscope Zeiss Discovery.V20 and photographed with a binocular microscope Olympus SZH10 with Nikon DS-Fi1 or the above-mentioned Zeiss microscope with a Canon EOS 70D camera. For the photographs of the external corallum surfaces specimens were coated with ammonium chloride. Photographs were arranged using digital image editing software. Descriptive terms follow Hill [18] and Zapalski [3]. Biometrical measurements were taken following Zapalski [3] and [19]; data on intracolonial variation follows the former. The specimens are housed at the University of Silesia, Faculty of Earth Sciences, Sosnowiec, Poland. Specimen numbers (acronymed GIUS) are given along with descriptions of taxa.

The electronic edition of this article conforms to the requirements of the amended International Code of Zoological Nomenclature, and hence the new names contained herein are available under that Code from the electronic edition of this article. This published work and the nomenclatural acts it contains have been registered in ZooBank, the online registration system for the ICZN. The ZooBank LSIDs (Life Science Identifiers) can be resolved and the associated information viewed through any standard web browser by appending the LSID to the prefix “http://zoobank.org/”. The LSID for this publication is: urn:lsid:zoobank.org:pub:B043831A-B8FF-44F0-8A83-FDA06AF5C599. The electronic edition of this work was published in a journal with an ISSN, and has been archived and is available from the following digital repositories: PubMed Central, LOCKSS.

## Geological Setting

**Fig 1:**
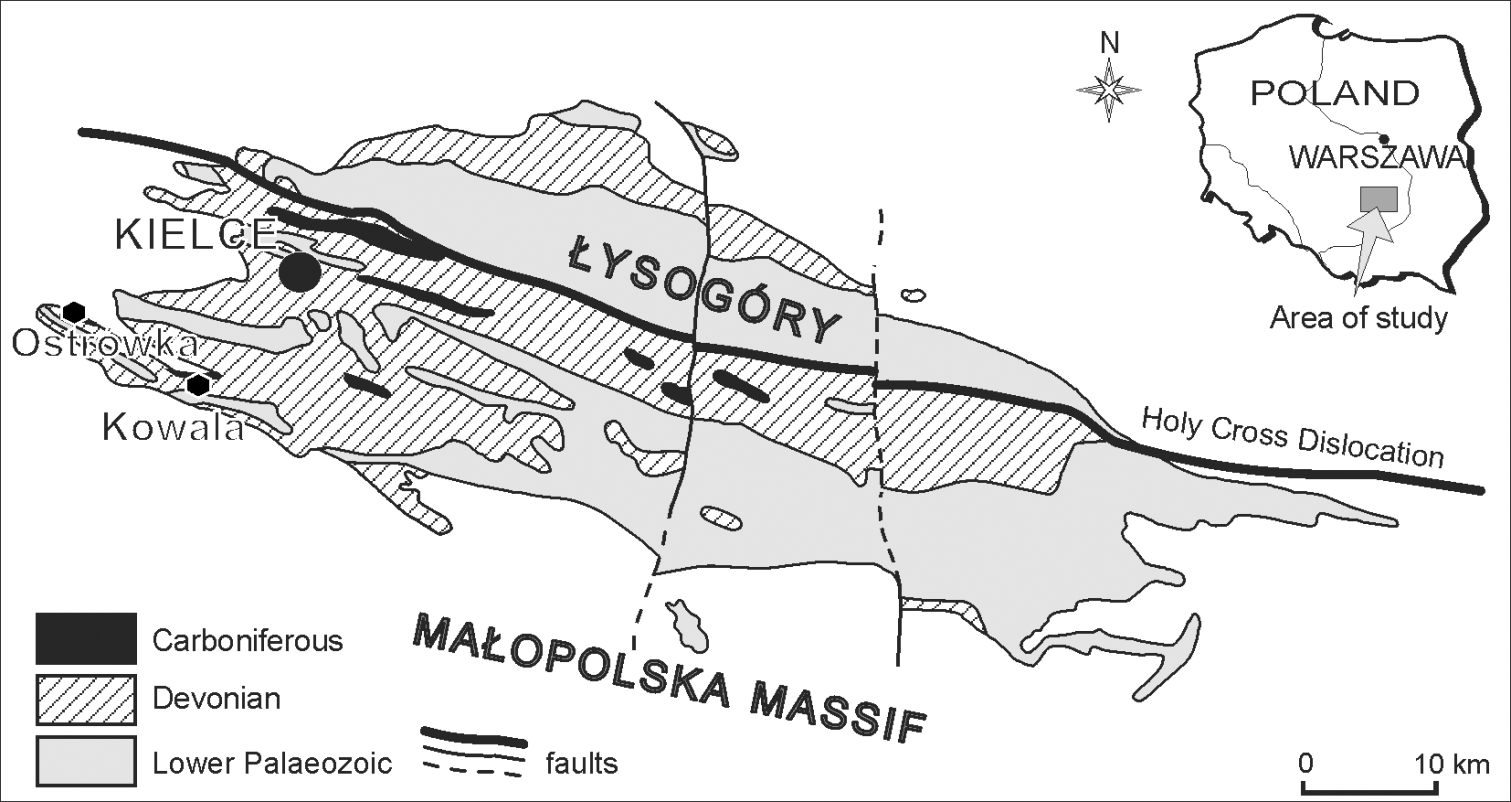
Simplified geological map of the Holy Cross Mts. Based on [23], modified and taken from [9] with Kowala and Ostrówka sites marked. Reproduced from [9] with permission from *Acta Geologica Polonica*.

The material analysed in the present study comes from two sites, located in the western part of the Holy Cross Mountains—the Kowala and Ostrówka Quarries (Fig 1). Kowala Quarry is located 10 km South from the center of Kielce, the main city in the region, while Ostrówka Quarry lies some 15 km SW of Kielce (Fig 1). Structurally both sites are located in the western part of the Kielce-Łagów Synclinorium, on the southern flank of Gałęzice Syncline.

Both Kowala and Ostrówka quarries exploit Devonian platform carbonates which developed in the Holy Cross area in the Givetian-Frasnian interval, at the subtropical SE margin of Euramerica [20, 21]; in both locations Famennian deposits represent the post-platform phase, with Kowala and Ostrówka representing respectively deeper and shallower sedimentation on drowned pre-Famennian carbonate platform. The depositional evolution of the whole basin was summarized by Szulczewski [22].

### Kowala

**Fig 2:**
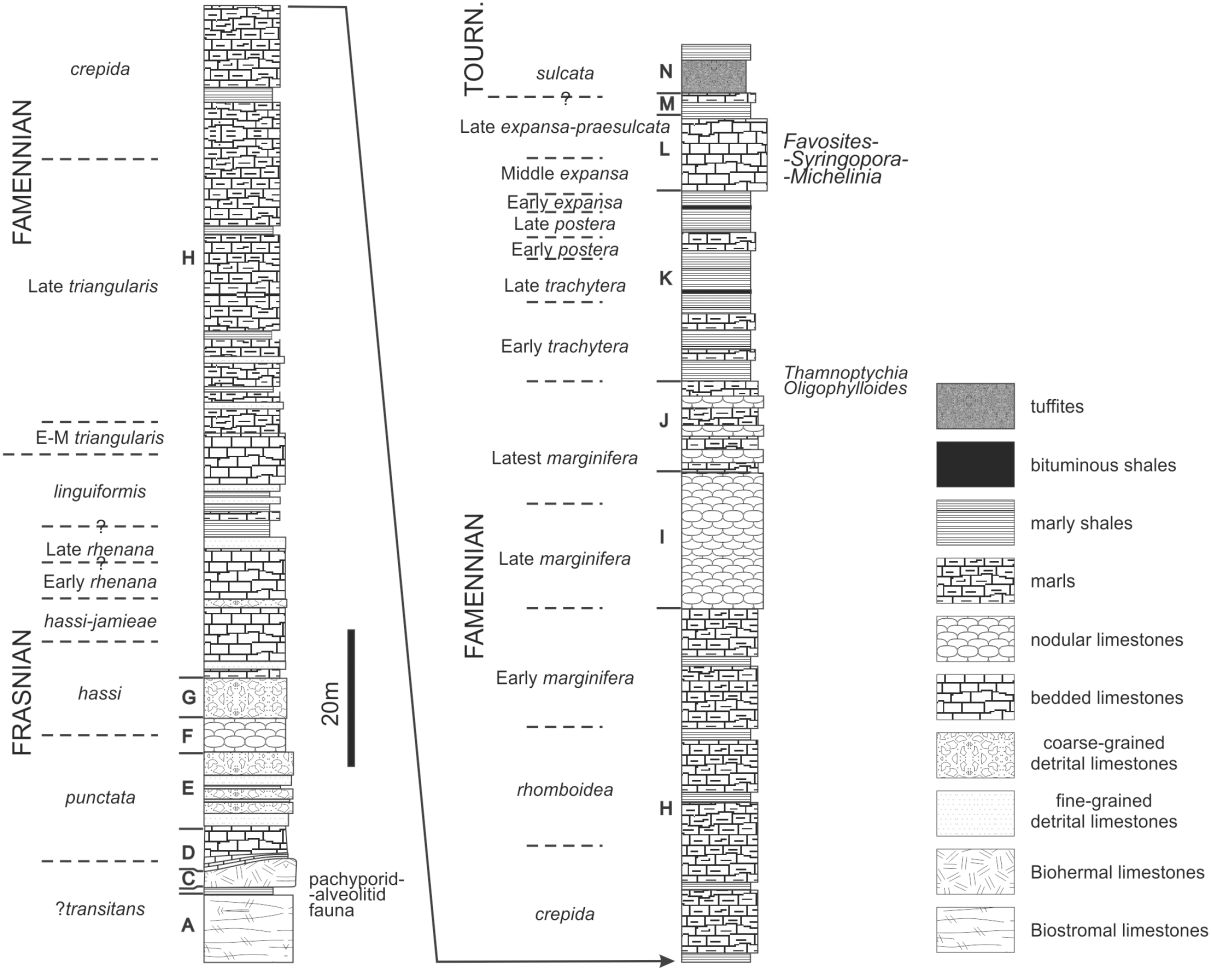
Simplified lithostratigraphic column of the Devonian in Kowala. Based on lithostratigraphic columns and data from various sources [3, 12, 26, 32], simplified.

The Kowala Quarry is situated south of Kielce (Fig 1). The age of the carbonate beds exposed in the Kowala quarry (and nearby Railroad Section) ranges from the Frasnian to the early Tournaisian [20, 23–25]. The whole Upper Devonian lithologic succession was divided by Szulczewski [26] and Berkowski [12] into twelve informal lithological sets A–L, with later additions (Fig 2). Sets A-G representing Frasnian carbonate deposits were described in detail by Szulczewski [23, 26]. Berkowski [12, 27] extended the section into the Famennian describing sets H-L cropping out in the Quarry. The Frasnian part of the section, with biostromal and biohermal limestones at the base, yielded abundant tabulate corals [3, 17, 28–30]; altogether eighteen species have been described from these beds. The Famennian part of the section consists of alternating limestones, marly limestones and shales [12], and records deepening of the basin [31].

The Famennian part of the whole sequence yielded locally abundant cephalopods, including clymeniids and goniatitids [33, 34]. Benthic faunas are represented by rugose corals, brachiopods, and trilobites [12, 27, 35, 36]. A single corallum of tabulate ?*Yavorskia paszkowskii* was described from the Upper Famennian of this location [9].

Famennian tabulate corals described in this paper were found at two stratigraphic levels. The lower tabulate bearing horizon is the uppermost part of the set J or the lowermost part of set K [12, 26, 27], and contains only a single species of branching tabulate, described below as *Thamnoptychia mistiaeni* sp. nov. accompanied here by the heterocoral *Oligophylloides*. These beds are represented by knobby dark limestones intercalating with black bituminous shales. They contain blind trilobites [27, 37]; pyritized goniatites and locally abundant *Guerichia* bivalves [12, 38]. Rugose corals are represented only by *Circiella concava*, solitary corals with lonsdaleoid vesicles [12]. The age of these beds is uppermost *Palmatolepis marginifera* to lowermost *Palmatolepis trachytera* [12].

The majority of the material described here comes from the bedded pelitic and partly nodular olive-green marly limestones (set L of [12, 26, 27]). The upper part of the set L corresponds to the lithological set (“complex”) A *sensu* Malec [24] and Marynowski and Filipiak [39], which is dated as Early *Palmatolepis expansa* to *Siphonodella praesulcata* Zones [see also: 9, 24, 40]. Most tabulate specimens were collected from the rubble of set L.

### Ostrówka

The large, active Ostrówka quarry near Gałęzice exposes a section from Frasnian to late Visean. The sedimentary history and complicated synsedimentary tectonics of this site have been described by Szulczewski [41] and Szulczewski *et al*. [31]. Five weathered fragments of branching tabulates described below as *Thamnoptychia mistiaeni* sp. nov. were found in the rubble originating probably from the condensed Famennian sequence (usually not exceeding 2 m in thickness), age of which has been determined as upper *marginifera* to lower *praesulcata* Zones [31]. As our specimens were found in the rubble, free of sediment, there is possibility that they may come from younger, Tournaisian rocks. From this site Różkowska [35] described the Famennian rugose coral *Pseudamplexus granulatus*, later interpreted as the tabulate coral *Actinotheca tenuicostata* [15]. Due to lack of further sedimentological data and scarcity of material, this site is not considered in further discussions on palaeocology and palaeoenvironment.

## Systematic Palaeontology

*Remarks*. Systematics here follows Hill [18] for Favositida and Zapalski [3] for Syringoporida and Auloporida. As a general rule, intracolonial variation has not been studied in previous papers dealing with Famennian tabulates, hence any comparisons of intracolonial variation are not possible.

Class: Anthozoa

Subclass: Tabulata Milne-Edwards & Haime, 1850

Order: Favositida Wedekind, 1937

Family: Favositidae Dana, 1846 (?)

Genus: *Favosites* Lamarck, 1816 (?)

?*Favosites* sp. 1

Fig 3A, 3B and 3C.

**Fig 3:**
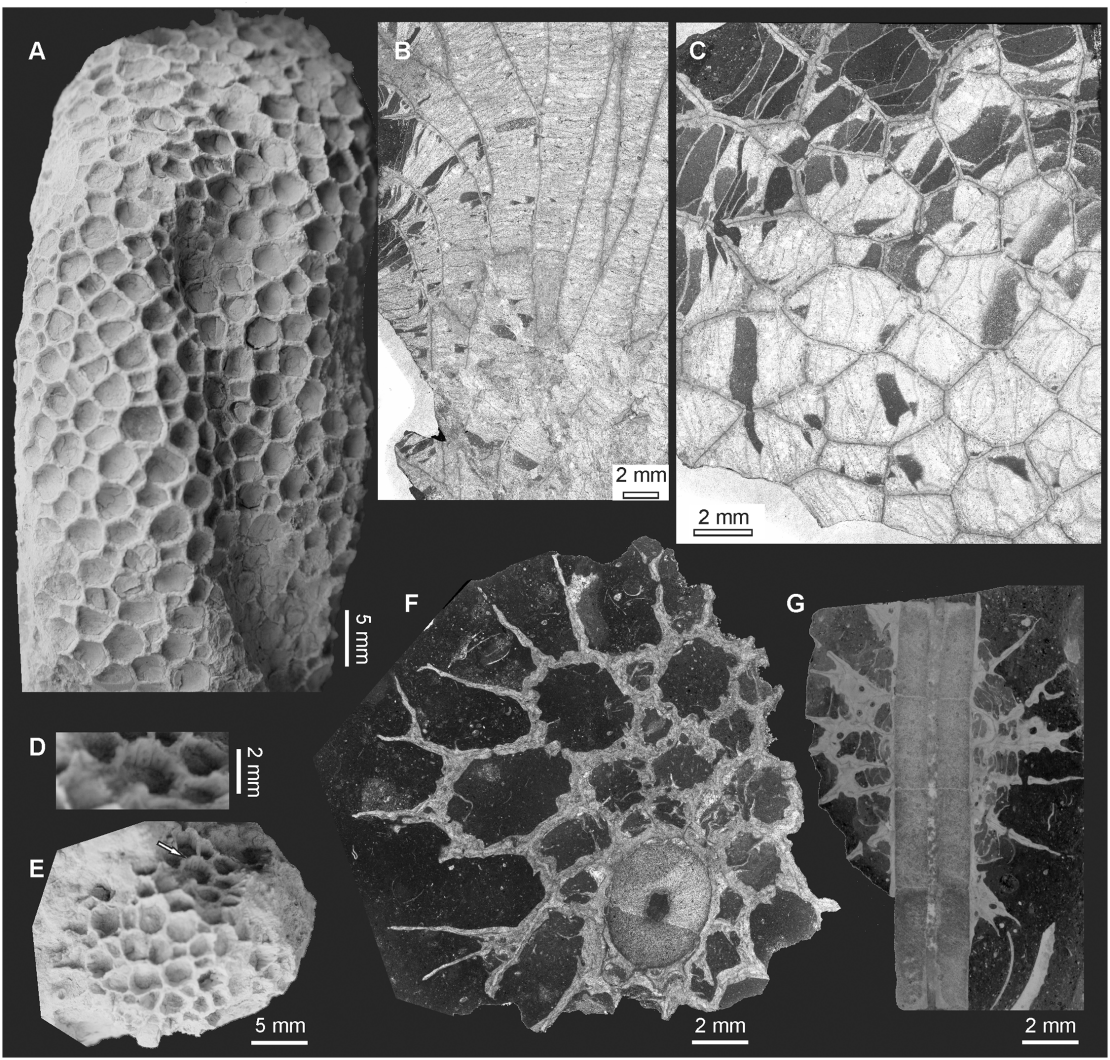
?*Favosites* from Kowala Quarry, lithological set L, late Famennian. (A) ?*Favosites* sp. 1, external view of the corallum. (B) *Favosites* (?) sp. 1, longitudinal section through the corallum. (C) ?*Favosites* sp. 1, transverse section through the corallum. Specimen GIUS 3619 KF 002. (D) and (E) ?*Favosites* sp. 2 external view of the corallum. (D) shows the detail of (E), where the arrangement of mural pores is visible. (F) ?*Favosites* sp. 2, transverse section through the corallum. Specimen GIUS 3619 KF 104 (G) ?*Favosites* sp. 2, longitudinal section through the corallum encrusting a crinoid. Specimen GIUS 3619 KF 091.

**Material:** Kowala Quarry, lithological set L, early *Palmatolepis expansa* to *Siphonodella praesulcata* Zones, late Famennian. Five coralla (GIUS 3619 KF 002, 003, 004, 101, 102), 12 thin sections.

**Description:** Coralla irregularly bulbous or oval, reaching about 80 mm in maximal diameter, with unknown attachment place. Corallites polygonal in cross section, calyces shallow, with thin, sharp edges. Smaller diameter of corallites (measured between opposite walls, from one median line to another) is 3.11 mm (mean; SD = 0,38 mm, N = 22). Tabulae thin, complete and incomplete, densely distributed, 15–25 per 5 mm, without periodicity of growth. Walls mostly even and smooth, only in distal parts of corallites somewhat uneven. Double wall thickness 0.28 mm (mean; SD = 0.09 mm, N = 22). Median line well visible. Septal spines absent. Mural pores round, located on peripheries of walls, about 0.20 mm in diameter. Their spacing cannot be observed on the material.

**Remarks:** The corallum structure, shape of corallites, placement of mural pores and shape of tabulae suggest assignment to the genus *Favosites*. The extremely large number of described species (nearly 900, our own estimation based on various sources) most of them probably invalid; coupled with very few available diagnostic characters, make any comparisons impossible. We therefore describe possible representatives of this genus in open nomenclature. Small coralla do not allow detailed study of intracolonial variation in this species.

The stratigraphic range of the genus *Favosites* ends most probably at the top of the Middle Devonian [18], but several species have been reported from the Carboniferous and Permian [42, 43]. *Favosites* (?) *stuarti* Nelson, 1962 from the Lower Permian of western Canada differs from the species described above by smaller corallites (up to 2.5 mm) and absence of mural pores [43]. The latter feature makes the generic attribution dubious.

As it is unlikely to have such a long duration of closely related species, it is possible that the appearance of "*Favosites*" morphology is rather an effect of convergence than a true evolutionary relationships between "*Favosites*" representatives from the entire Ordovician to Permian interval.

?*Favosites* sp. 2

**Fig 4:**
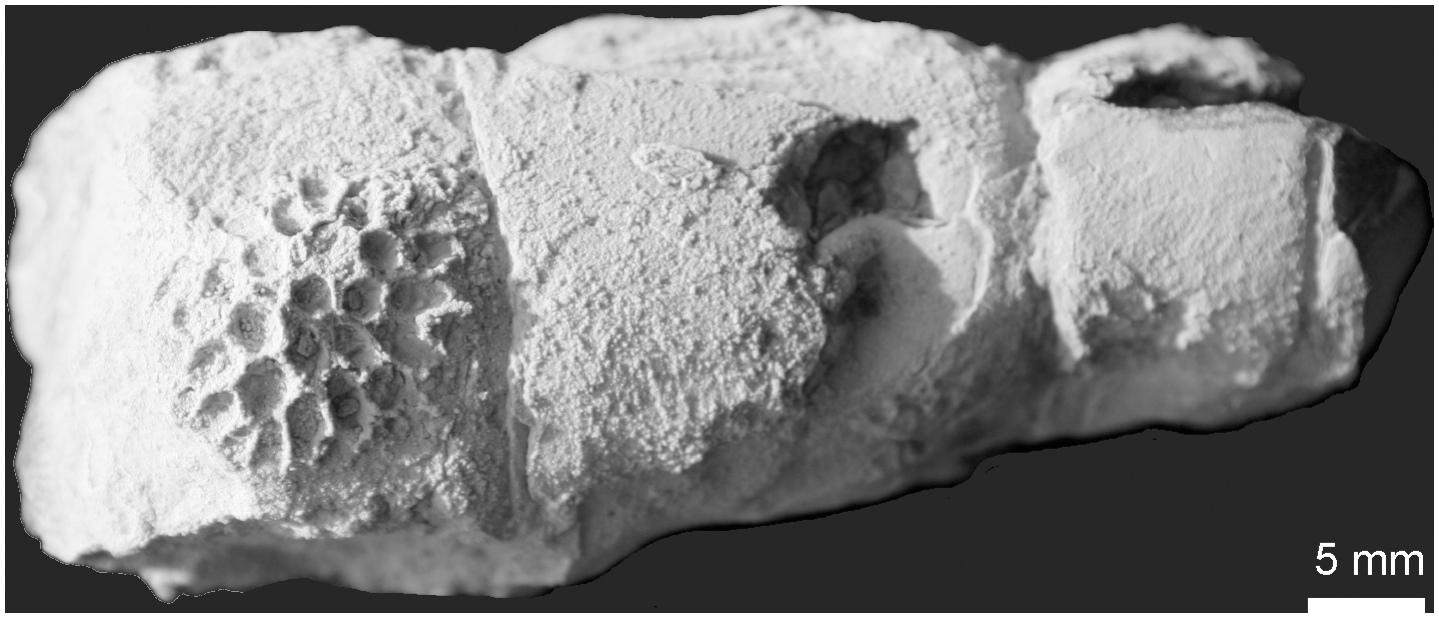
?*Favosites* sp. 2 encrusting nautiloid, from Kowala Quarry, lithological set L, late Famennian. Specimen GIUS 3619 KF 092.

Figs 3D, 3E, 3F and 4.

**Material:** Kowala Quarry, lithological set L, early *Palmatolepis expansa* to *Siphonodella praesulcata* Zones, late Famennian. Three coralla (GIUS 3619 KF 091, 092 and 104), five thin sections.

**Description:** Coralla close to round, small (about 15mm in maximum diameter), encrusting fragments of crinoid stem. Corallites polygonal in cross section, calyces about twice as deep as wide, with thin, sharp edges. Smaller diameter of corallites 2.0–2.7 mm. Tabulae thin, complete and incomplete. Walls strongly uneven, crenulated in cross section, with irregularly distributed, septal spines. Septal spines highly variable in shape and size, most often sharply pointed, 0.25–0.70 mm in length. Few mural pores probably located near the median of the walls, round, arranged in rows, about 0.10–0.12 mm in diameter, with unknown spacing.

**Remarks:** The generic assignment of these specimens is doubtful, as they display features of several genera. Some incomplete tabulae may be longitudinal sections through squamula-like structures, resembling those of the genus *Hamarilopora* (subfamily Emmonsiinae). The distribution of mural pores cannot be observed in detail (this feature is distinctive for several genera), and these "squamulae" are observed only in a few corallites, so their generic significance must remain dubious. This species may also slightly resemble *Sutherlandia* (also Emmonsiinae), but in *Sutherlandia* tabulae are lacking [18]. Small coralla and small number of corallites do not allow collection of sufficient data for biometrical study, therefore only approximate ranges are given. The species described above as ?*Favosites* sp. 1. has larger coralla, non-crenulate walls, and lacks septal spines.

Family Alveolitidae Duncan, 1872 (?)

?Alveolitidae indet.

Fig 5

**Fig 5:**
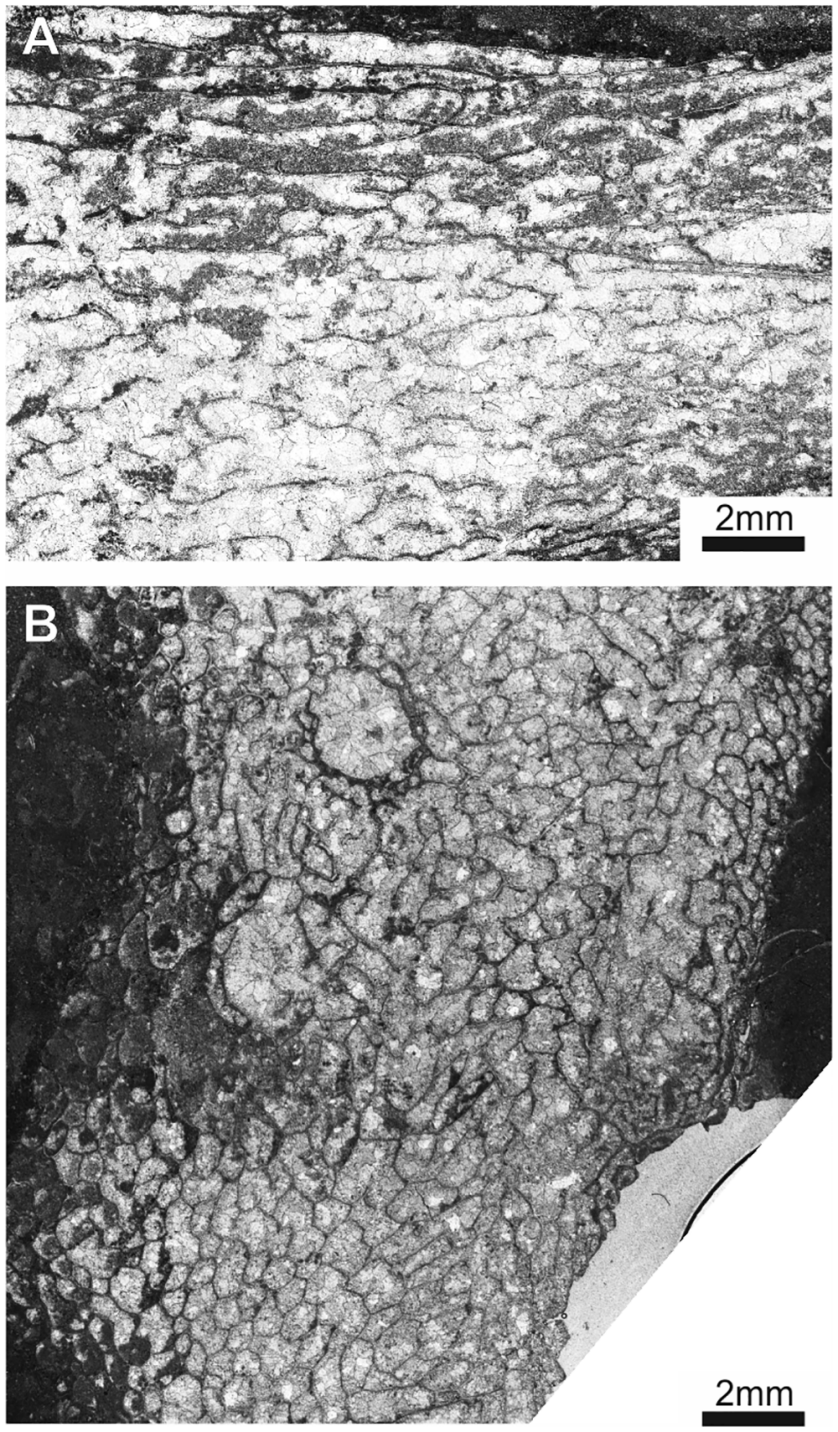
?Alveolitidae indet. from Kowala Quarry, lithological set L, late Famennian. (A) section close to longitudinal. (B) section close to transverse. Specimen GIUS 3619 KK 251.

**Material:** One incomplete fragment of corallum (GIUS 3619 KK 251), lithological set L, early *Palmatolepis expansa* to *Siphonodella praesulcata* Zones, late Famennian. Three thin sections.

**Description:** Corallum tabular, analyzed fragment measures about 50 × 50 × 30 mm. Corallites variable in cross sections, from polygonal to elongated and irregular, probably curved. Their diameter is usually around 0.5 mm. In the analyzed fragment no correct longitudinal section was obtained, and the angle of corallites reaching the surfae is unknown. Walls uniformly thin, rare septal spines are short and sharp. Mural pores in some regions so numerous that wall structure becomes cribriform and the whole corallum has spongy appearance, in other regions nearly absent. Tabulae probably absent (no longitudinal section obtained).

**Remarks:** This enigmatic fossil resembles alveolitids (such as *Spongioalveolites* Iven, 1980), but small diameters and even, thin walls may even suggest bryozoan affinity. Due to complicated geometry of corallites and lack of correctly oriented sections detailed biometrical study and more accurate determination could not be obtained.

Family Micheliniidae Waagen and Wentzel, 1886

Genus: *Michelinia* de Koninck, 1841

?*Michelinia vinni* sp. nov.

urn:lsid:zoobank.org:act: 54CCC26C-AE04-49AA-BE63-FEF49EA99398

Fig 6

**Fig 6:**
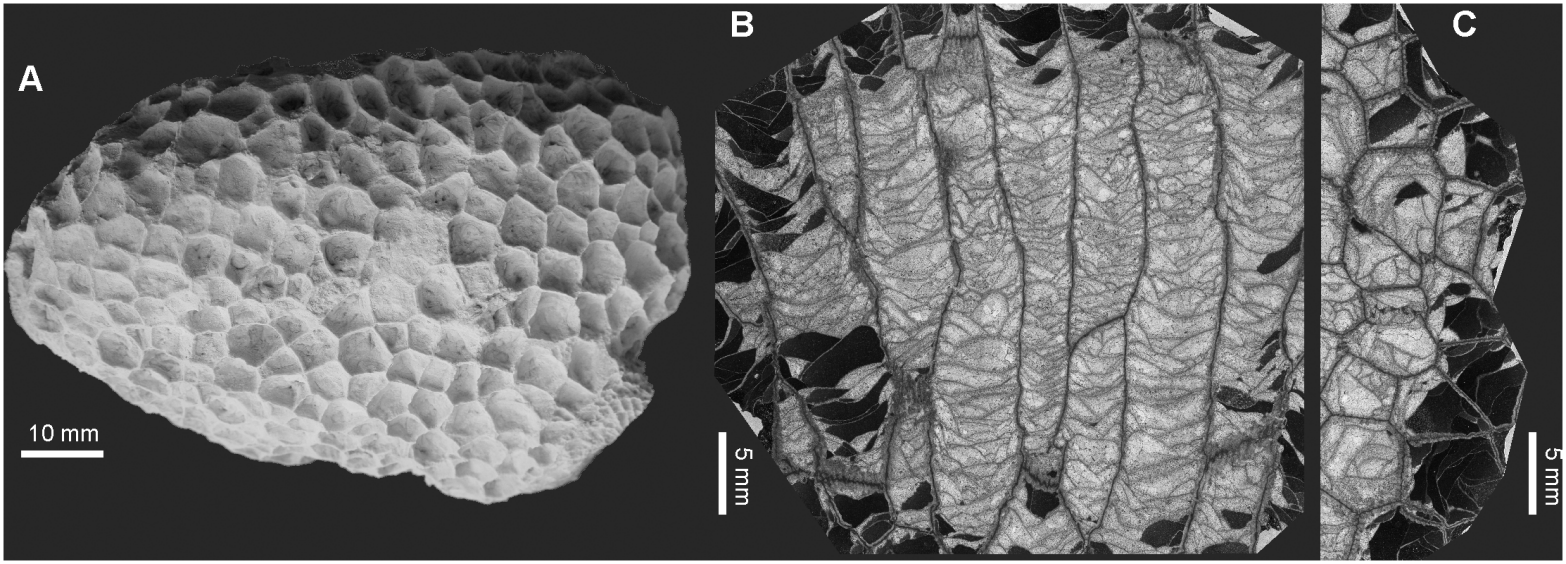
?*Michelinia vinni* sp. nov. from Kowala Quarry, lithological set L, late Famennian. (A) External view of the corallum. (B) Longitudinal section. (C) Transverse section. Specimen GIUS 3619 KF 103, holotype.

**Derivation of the name:** In honour of Dr Olev Vinn (Tartu University, Estonia), palaeontologist, for his contribution to knowledge of ecology of Palaeozoic bioconstructing organisms.

**Material:** One corallum (holotype, GIUS 3619 KF 103), Kowala Quarry, lithological set L, early *Palmatolepis expansa* to *Siphonodella praesulcata* Zones, late Famennian. Three thin sections.

**Type stratum:** Lithological set L, Early *Palmatolepis expansa* to *Siphonodella praesulcata* Zones, upper Famennian.

**Type locality:** Kowala Quarry, Holy Cross Mountains, Poland.

**Diagnosis:** Corallum small, massive. Mean calyx diameter 5.7 mm, corallite diameters range from 5.0 to 6.3 mm. Tabulae variable in shape, mostly incomplete, septal spines rare.

**Description:** Corallum cerioid, irregular, measuring 52 × 48 × 80 mm. Corallites grow at a right angle to the corallum surface at the upper surface, and at varying angles on the sides. Calyces polygonal, shallow (wider than deeper), with depth usually 2–3 mm. Corallites long, reaching about 50 mm in length, prismatic, polygonal in cross section. Corallite walls thin, uneven. Median suture well visible throughout the corallum. Mural pores small, round. Septal apparatus poorly developed, nearly absent. Broad (about thrice as broad as long), short and blunt, somewhat button-like septal spines can be regarded rather as internal irregularities of the wall. Tabulae numerous, mostly incomplete, concave, flat, convex, sometimes bubble-convex, without growth periodicity. Biometrical data are given in the Table 1.

**Table 1:** Intracolonial variation in ?*Michelinia vinni* sp. nov.

Specimen GIUS 3619 KF 103, holotype				
	Calyx diameter	Maximal corallite diameter	Double wall thickness	Pore diameter
mean [mm]	5.71	-	0.37	-
standard dev. [mm]	0.53	-	0.12	-
N	18	4	17	6
minimal value [mm]	4.7	5.0	0.2	0.1
maximal value[mm]	6.8	6.3	0.6	0.2
*V*	0.093	-	0.32	-

**Remarks:** The species described here is very similar to the Carboniferous *Michelinia tenuisepta* (Phillips, 1836), especially in terms of biometry (see: [13], p. 48), but the new species differs in absence of spines on the tabulae and somewhat smaller diameters of coralla (6–10 mm in *M*. *tenuisepta* [13]). *Michelinia guerangeri* (Milne-Edwards et Haime, 1851) has much smaller corallites (3.8–4.5 mm; see [44] and references therein). Three species of *Michelinia*, known from the earliest Carboniferous of Omolon Region, are also different. Both *Michelinia lacunosa* Smirnova, 1984 and *M*. *costata* have much larger corallites, with diameters from 8 to 11 mm (*M*. *costata*) and 10 to 14 mm (*M*. *lacunosa*). *M*. *catenata* Smirnova, 1984 from the same beds has corallites ranging from 6.0 to 9.5 mm and the colony structure is cerioid-chain like, with formation of lacunae (Smirnova in [45]). *M*. *harkeri* Nelson, 1962 from the Upper Pennsylwanian of western Canada is larger, with corallite diameters between 7 and 13 mm [43].

The intracolonial variation of calyx diameter is very low, as compared to Frasnian tabulates, such as *Alveolites* or *Crassialveolites*, and much more similar to that of some Silurian heliolitids [46].

Family: Pachyporidae Gerth, 1921

Genus: *Thamnoptychia* Hall, 1876

**Remarks:** The systematic position of this genus is unclear. Its affinities have been discussed both on the level of skeletal structures [47] and microstructures [48]. The latter author proposed creation of a separate family, Thamnoptychiidae Lafuste, 1988 for *Thamnoptychia* and *Dendropora*. Such systematic assignment is based solely on microstructural features. As there is no consensus regarding the importance of microstructure for pachyporid taxonomy, and there are not enough data concerning pachyporid microstructure, *Thamnoptychia* is placed in the Pachyporidae, following Hill [18]. The diagnosis of this genus was given by Tourneur ([47] p. 428). The genus is probably monospecific.

*Thamnoptychia mistiaeni* sp. nov.

urn:lsid:zoobank.org:act: CDDBB602-C859-4C4F-8AAA-109E436E8693

Figs 7, 8 and 9

**Fig 7:**
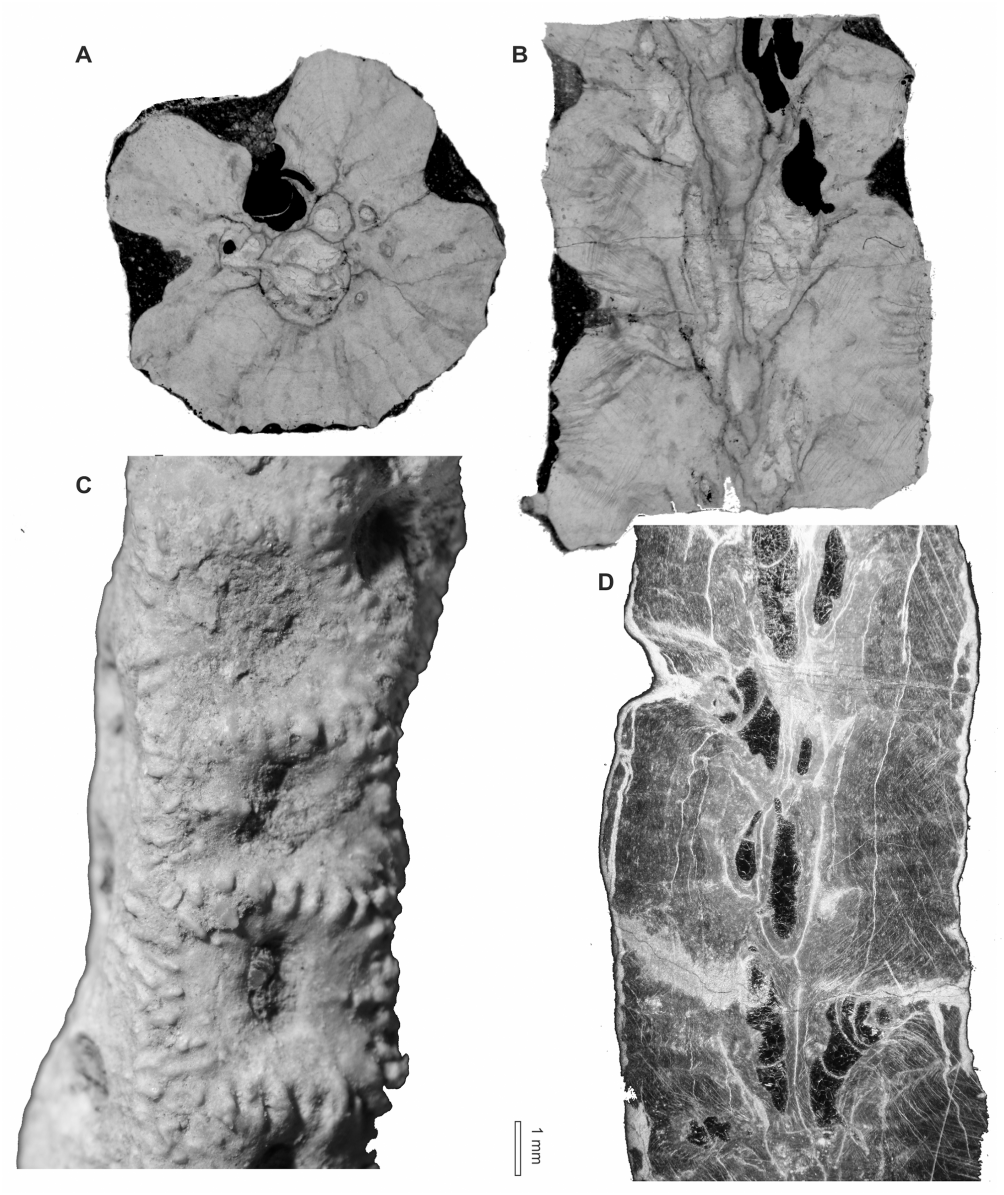
*Thamnoptychia mistiaeni* sp. nov. from Kowala Quarry, lithological sets J or K, Upper Famennian. (A) Transverse section through a branch. (B) Longitudinal section through a branch. Specimen GIUS 3619 KF 049, holotype. (C) External view of a branch. Specimen GIUS 3619 KF 090a. (D) Longitudinal section through a branch. Specimen seen in dark field, GIUS 3619 KF 090b.

**Fig 8:**
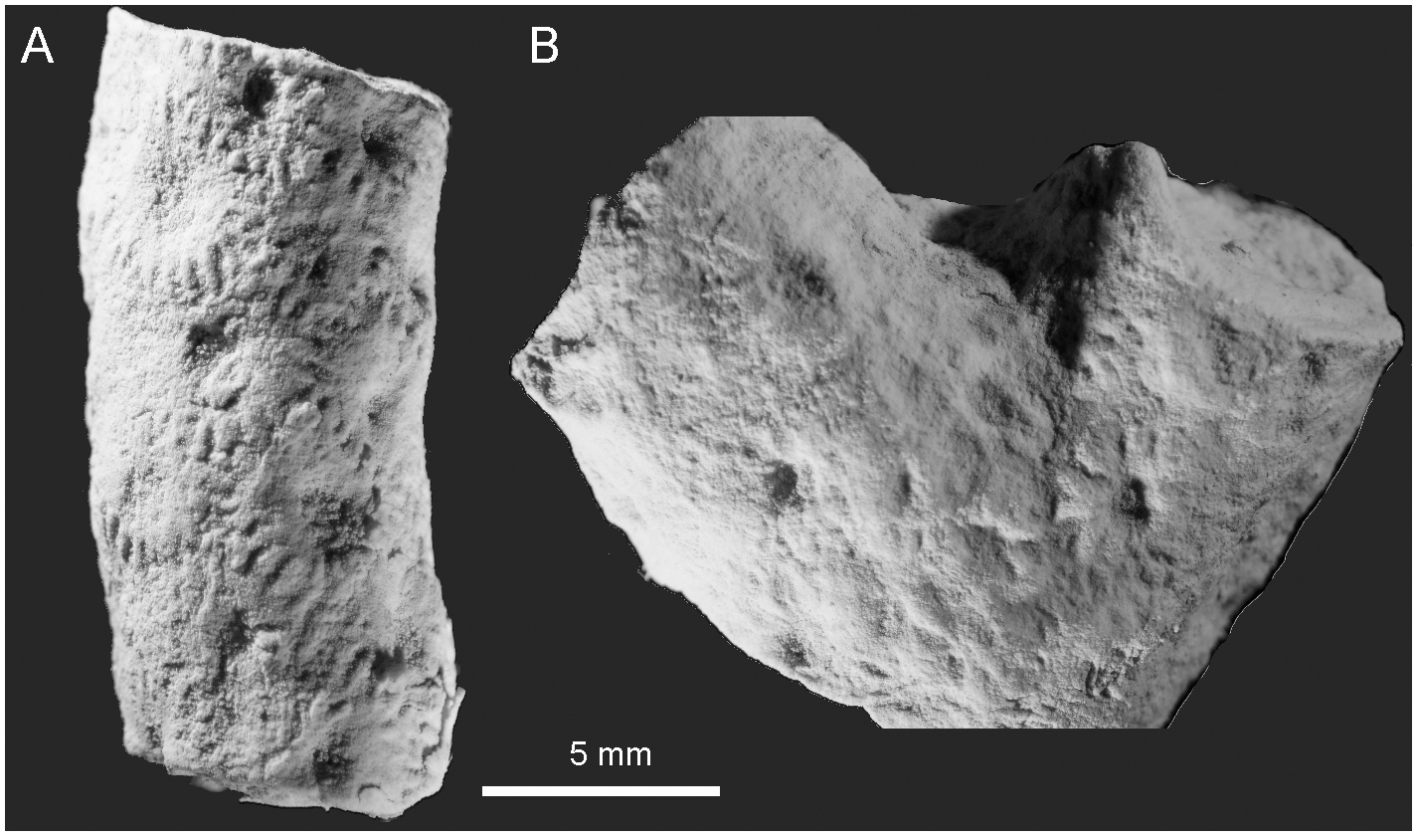
*Thamnoptychia mistiaeni* sp. nov. from Kowala Quarry, lithological sets J or K, Upper Famennian. (A) External view of a branch. Specimen GIUS 3619 KF 090c. (B) External view of a branch. Specimen GIUS 3619 KF 090d.

**Fig 9:**
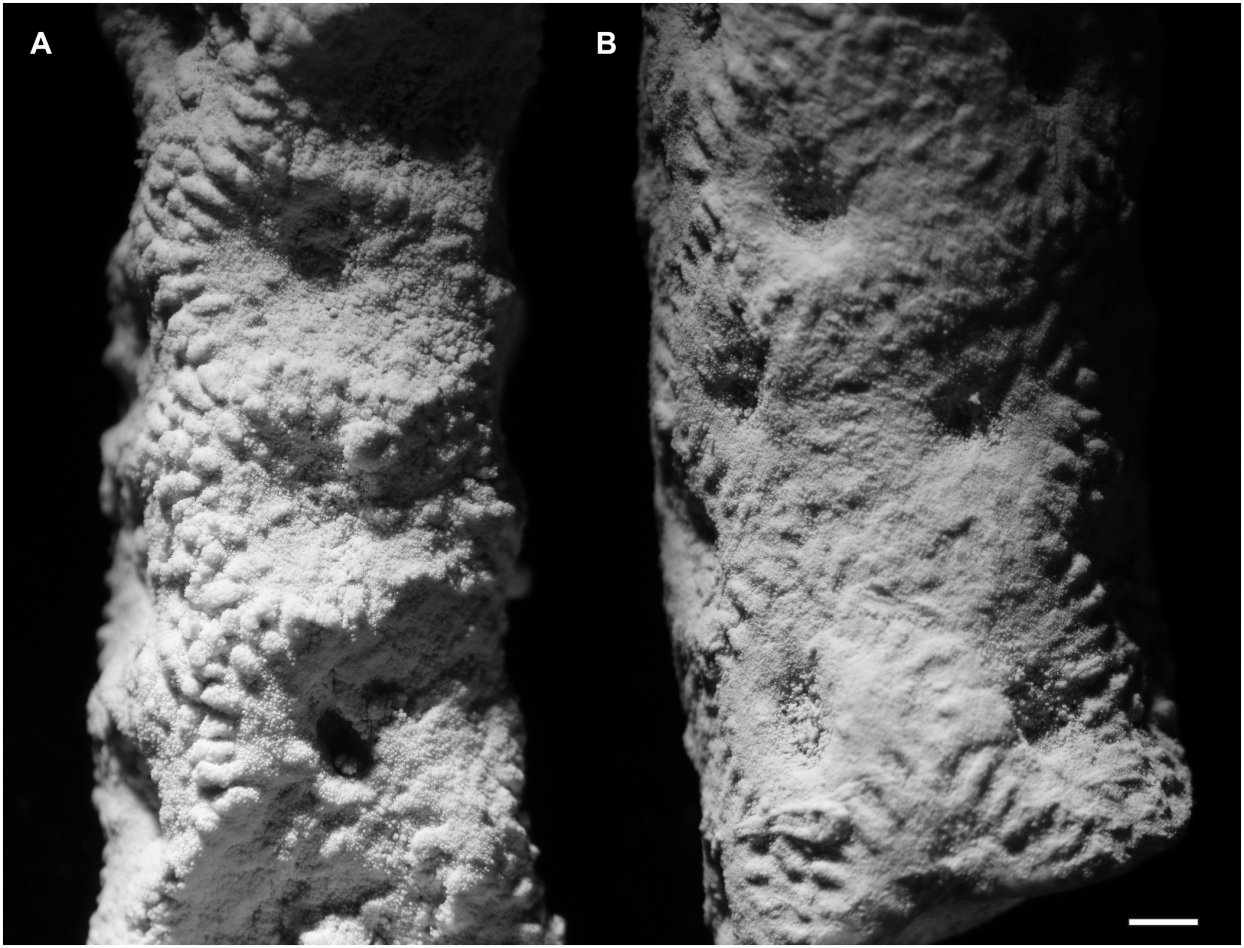
*Thamnoptychia mistiaeni* sp. nov. from Kowala Quarry, lithological sets J or K, Upper Famennian. (A) External view of a branch, specimen GIUS 3619 KF 090a (same as Fig 7C). (B) External view of a branch, specimen GIUS 3619 KF 090c (same as Fig 8A). Notice polygonal (subrectangular to subhexagonal) shape of calyces.

**Derivation of the name:** In honour of Professor Bruno Mistiaen (Université Catholique de Lille, France), for his contribution to knowledge of the Late Famennian.

**Material:** Holotype: Specimen GIUS 3619 KF 049; paratypes: altogether 26 broken branches (collectively under numbers GIUS 3619 KF 089, 090) from Kowala Quarry, lithological set J or K, late *Palmatolepis marginifera* to lower *P*. *trachytera* Zones, Upper Famennian; seven thin sections, nine acetate peels. Five broken branches from Ostrówka (collective sample GIUS 3630 OF 004), possibly upper Famennian, five acetate peels.

**Type stratum:** Upper Famennian, lithological sets J or K of Kowala Quarry, late *Palmatolepis marginifera* to lower *P*. *trachytera* Zones

**Type locality:** Kowala Quarry, Holy Cross Mountains, Poland.

**Diagnosis:** *Thamnoptychia* with rare septal spines and lacking squamulae.

**Description:** Corallum branching. Branches from 4 to 12 mm in diameter. Calyces shallow, widely conical, with presence of radiating ornament of external parts of stereozone, 3.0 to 4.5 mm in diameter. Corallites polygonal in cross section, reaching the corallum surface at the angle close to 60°. Calyces polygonal (often subpolygonal), from four- to six sided. Walls thin in the axial zones of branches, thickening significantly in the distal zones, and occypying about 60–70% of diameter in distal zones. Intercorallite suture poorly visible. Septal apparatus developed as very rare spines (Fig 7A)–long, thin and sharp, somewhat curved (note that only a single septal spine was observed). Tabulae not numerous, complete, in the form of concave meniscs. Mural pores not observed.

**Remarks:** The generic attribution of our material relies on the strong thickening of the walls and the presence of radiating ornament in the external parts of the stereozone. Many species of genera *Thamnoptychia* and *Trachypora* have been described, but it seems that they are all conspecific [47]. The type species, *T*. *limbata* is characterized by presence of numerous septal spines and squamulae (absent in small branches), numerous tabulae and strongly uneven walls [47]. The species described above is somewhat similar to *T*. *limbata*, but differs in absence of squamulae, less abundant tabulae and even walls. The type species has also irregularly distributed mural pores ([47]: figs. 209, 211, 212), not observed in our material. Due to scarce biometrical data it is difficult to asses the intracolonial variation, but it seems that branch diameter is the most variable parameter. On the other hand these are fragments collected from the rubble and original number of colonies is unknown.

**Fig 10:**
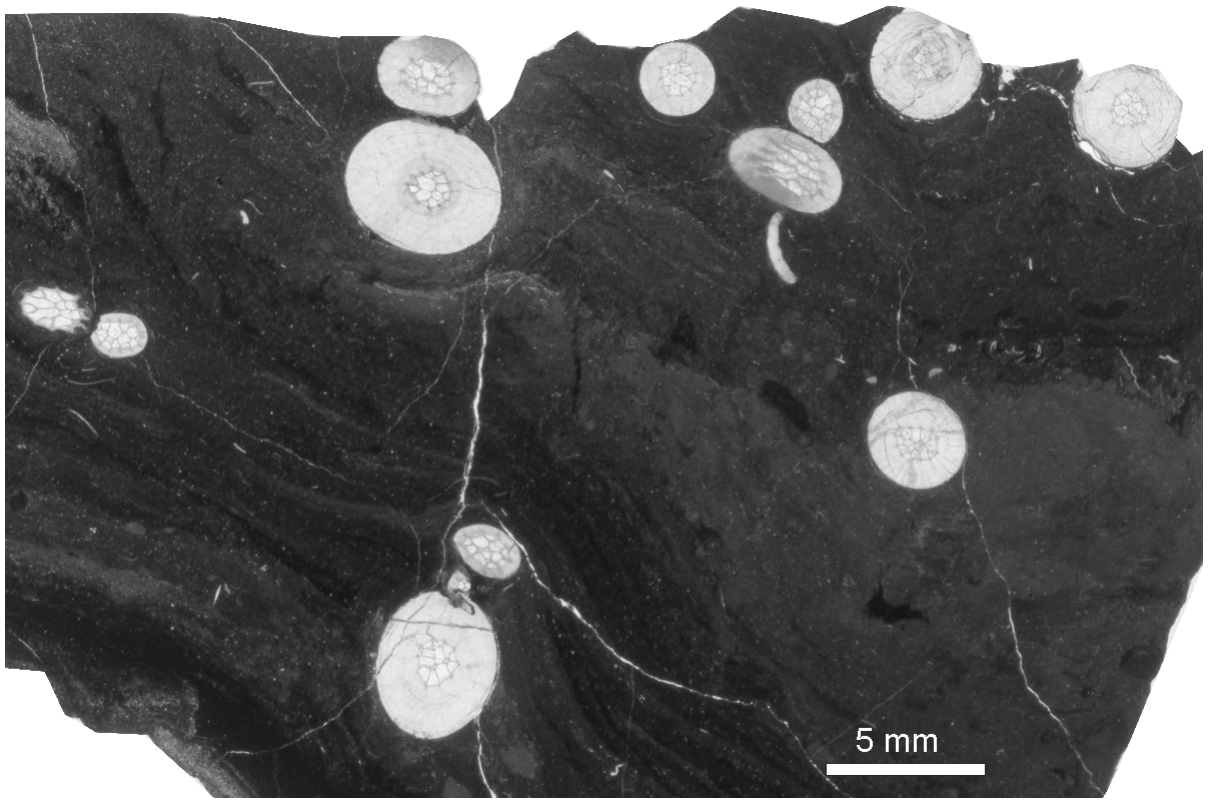
Heterocorals *Oligophylloides* co-occurring with *Thamnoptychia mistiaeni* sp. nov. Transverse sections through branches. Kowala Quarry, lithological sets J or K, Upper Famennian.

As mentioned above, this species occurs in the beds of lithological sets J or K, co-occuring with the heretocoral *Oligophylloides* (Fig 10).

Order: Syringoporida

Family Syringoporidae de Fromentel, 1861

Genus: *Syringopora* Goldfuss, 1826

*Syringopora kowalensis* sp. nov.

urn:lsid:zoobank.org:act: 56618416-12DC-446B-AE18-85791477341D

Fig 11

**Fig 11:**
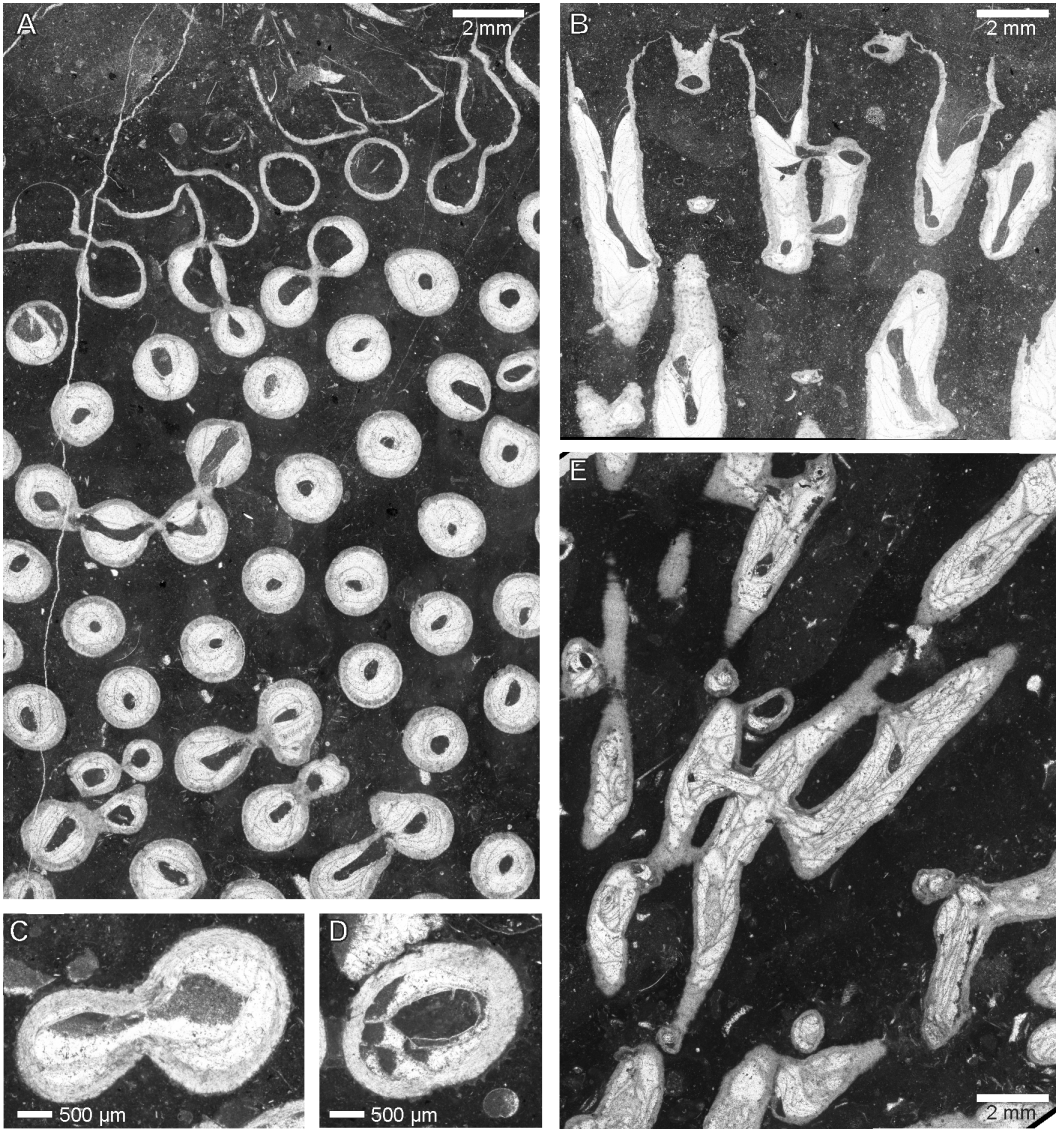
*Syringopora kowalensis* sp. nov. from Kowala Quarry, lithological set L, Upper Famennian. (A) Transverse section. (B) Longitudinal section. Notice deep calyces. Specimen GIUS 3619 KF 005, holotype. (C) and (D) Details of corallites. Specimen GIUS 3619 KF 008. (E). Longitudinal section. Specimen GIUS 3619 KF 009.

**Derivation of the name:** From the name of the type locality.

**Material:** Holotype: GIUS 3619 KF 005, paratypes: two coralla (GIUS 3619 KF 008 and 009) from Kowala Quarry, lithological set L, early *Palmatolepis expansa* to *Siphonodella praesulcata* Zones, upper Famennian. 17 thin sections

**Type stratum:** Upper Famennian, lithological set L of Kowala Quarry, early *Palmatolepis expansa* to *Siphonodella praesulcata* Zones,

**Type locality:** Kowala Quarry, Holy Cross Mountains, Poland.

**Diagnosis:** *Syringopora* with corallites with a mean diameter of 1.76 mm and a mean wall thickness of 0.20 mm. Tabulae complete and incomplete. Syrinx well developed, abundant septal and tabular spines and scarce connecting tubes.

**Description:** Small fragments of phaceloid coralla, largest measuring 50×40×30 mm. Corallites usually straight, tubular, round or slightly oval in cross section. Calyces deep, funnel -shaped, elongated, with sharp edges and numerous septal spines. Distances between corallites from 0.5 to 1.5 mm. Tabulae thin, complete or incomplete (tabellae), strongly bent downwards, bearing spines, and forming a deep axial canal (syrinx). Axial canal round, oval or lens shaped, rarely irregular in cross section, placed axially. Thickness of axial canal walls similar to thickness of tabulae. Walls usually even and smooth, but with numerous septal spines; near spines walls often uneven. Septal spines abundant, long and thin, with pointed ends. Tabular spines similar in shape and size to septal spines. Connecting tubes scarce, distributed irregularly, attaining 0.50 mm in diameter. Lumen of axial canal, round or oval, rarely irregular is often connected with lumen of connecting tubes. Its diameters are larger in more distal parts of corallites. Wall microstructure lamellar, with lamellae strongly bent around spines. Biometrical data are given in the Tables 2 and 3.

**Table 2:** Intracolonial variation in *Syringopora kowalensis* sp. nov.

Specimen GIUS 3619 KF 005, holotype.				
	Corallite diameter	Wall thickness	Septal spine length	Syrinx diameter
mean [mm]	1.757	0.195	0.125	0.401
Std. dev. [mm]	0.171	0.04	0.014	0.084
N	50	40	5	30
min. value [mm]	1.184	0.122	0.104	0.239
Max. value[mm]	2.035	0.296	0.146	0.571
*V*	0.097	0.205	0.112	0.209

**Table 3:** Intracolonial variation in *Syringopora kowalensis* sp. nov.

specimen GIUS 3619 KF 009, paratype.				
	Corallite diameter	Wall thickness	Septal spine length	Syrinx diameter
mean [mm]	1.991	0.272	125	-
Std. dev. [mm]	0.227	0.038	14	-
N	50	40	5	7
min. value [mm]	1.55	0.184	104	0.30
Max. value[mm]	2.499	0.365	146	0.50
*V*	0.114	0.140	0.112	-

**Remarks:** The discussed species is similar to *Syringopora longospina* Tchudinova 1970 from the Visean of Transcaucasia, from which it differs by much smaller diameters of connecting tubes (0.8–1.2 mm in *S*. *longospina*). Also septal spines in *S*. *longospina* are much longer (up to 0.6 mm), and denser [49]. The widespread lower Carboniferous *Syringopora reticulata* Goldfuss, 1826 is also similar to the new species, but it has axial canals larger in diameter (0.6–0.9 mm) and larger diameters of connecting pores (0.7–0.8 mm [13]). The only species of *Syringopora* occurring in the Famennian of the Holy Cross Mountains, *S*. cf. *volkensis* Tchernychev, 1938 differs by much smaller corallite diameters (mean: 1.33 mm, maximal value 1.54 mm), deeply infundibuliform tabulae and much thicker axial canal (0.54 to 0.66 mm), the wall thickness is in both species similar. The new species has probably wide intraspecific (extracolonial) variation, and two specimens described above slightly differ by biometrical features. The overall similar appearance of both coralla allows assigning them to one species.

The intracolonial variation of corallite diameters and wall thickness is low, as compared to that of alveolitids [3] and comparable with that of some heliolitids [50]. Wall thickness is also less variable than in alveolitids, yet it may reach *V* close to 0.2. On the other hand septal spine length is rather stable, with *V* values close to 0.1. Axial canal diameters are moderately variable.

*Syringopora hilarowiczi* sp. nov.

urn:lsid:zoobank.org:act: 252DE649-5F0A-41BB-98E4-13C2809A6F6E

Figs 12 and 13

**Fig 12:**
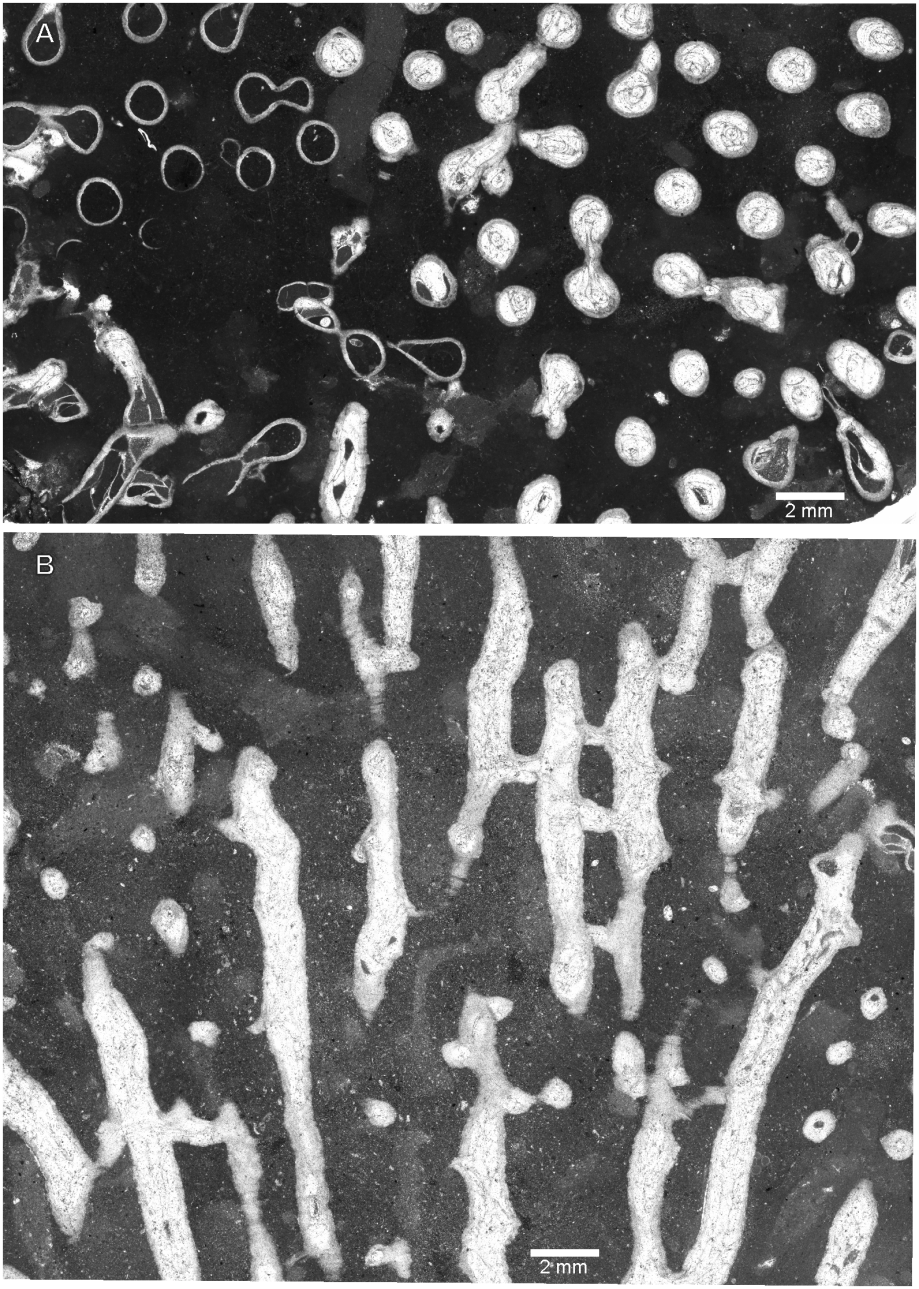
*Syringopora hilarowiczi* sp. nov. from Kowala Quarry, lithological set L, Upper Famennian. (A) Transverse section. (B) Longitudinal section. Specimen GIUS 3619 KF 006, holotype.

**Fig 13:**
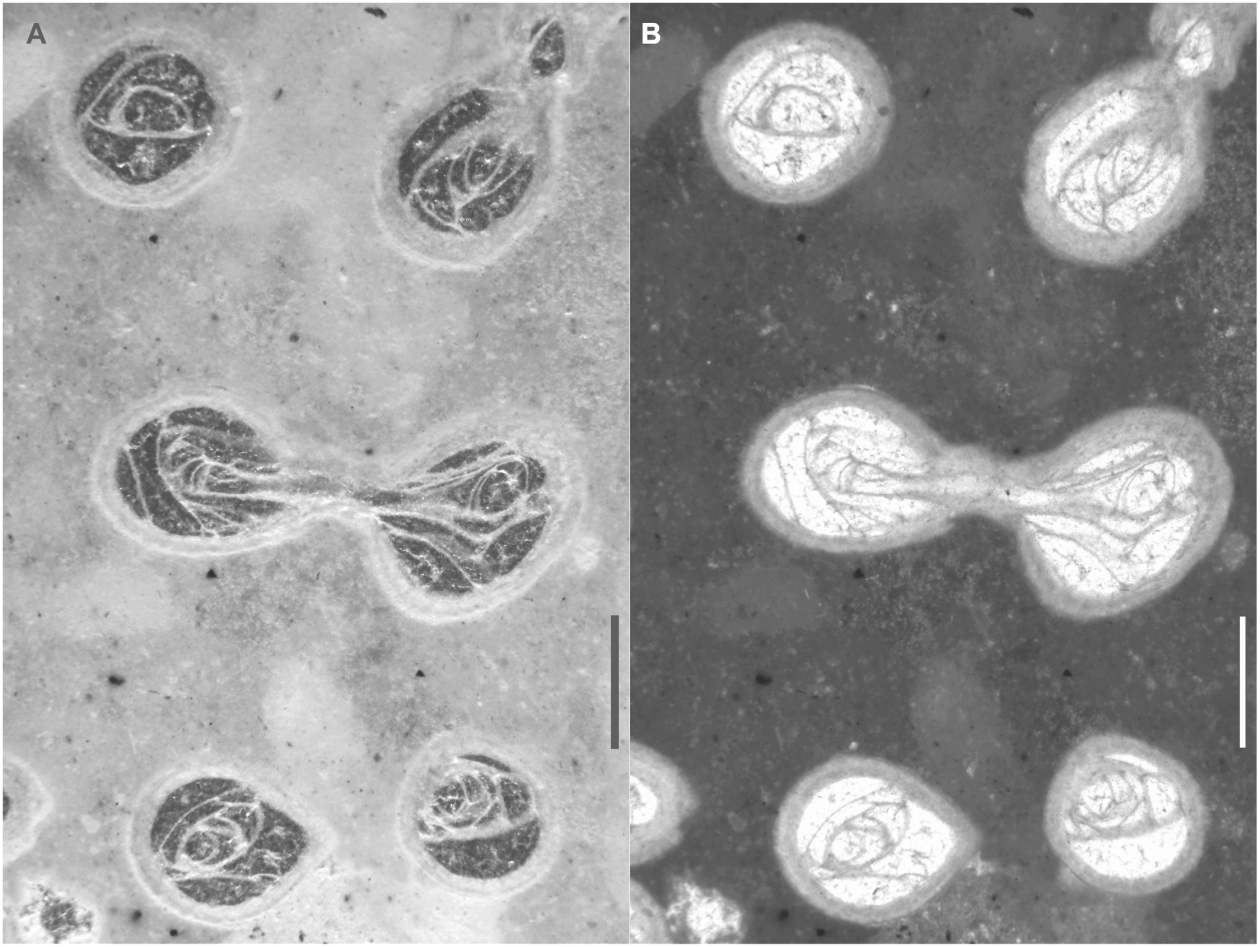
*Syringopora hilarowiczi* sp. nov. from Kowala Quarry, lithological set L, Upper Famennian. (A, B) Details of transverse sections. (A) Transmitted light. (B) Same section, dark field. Specimen GIUS 3619 KF 006, holotype.

**Derivation of the name:** In honour of Józef Nusbaum-Hilarowicz (1859–1917), zoologist.

**Material:** One incomplete corallum (GIUS 3619 KF 006, holotype), from Kowala Quarry, lithological set L, early *Palmatolepis expansa* to *Siphonodella praesulcata* Zones, upper Famennian. Six thin sections

**Type stratum:** Upper Famennian, lithological set L of Kowala Quarry, early *Palmatolepis expansa* to *Siphonodella praesulcata* Zones.

**Type locality:** Kowala Quarry, Holy Cross Mountains, Poland.

**Diagnosis:** Syringopora with corallites with an average diameter of 1.26 mm and an average wall thickness of 0.16 mm. Tabulae complete or incomplete. Syrinx present, rare septal and tabular spines and scarce connecting tubes.

**Description:** Medium-sized phaceloid corallum, measuring about 50 mm in diameter. Corallites straight, tubular, round in cross section. Calyces deep with sharp edges. Distances between corallites variable, from 0.5 to 1.7 mm. Tabulae thin, complete or incomplete, strongly bent downwards, possesing scarce spines, and often forming an axial canal (syrinx). Thickness of axial canal walls similar to thickness of tabulae. Syrinx irregular in cross section, placed axially or subaxially, in rare cases in contact with corallite wall. Walls usually even and smooth, with septal spines; near spines often uneven. Septal spines not numerous, moderately long and thin, with sharp terminations. Tabular spines rare, similar in shape and size. Connecting tubes scarce, distributed irregularly. Wall microstructure lamellar. The biometrical data is given in the Table 4.

**Table 4:** Intracolonial variation in *Syringopora hilarowiczi* sp. nov.

Specimen GIUS 3619 KF 006, holotype.		
	Corallite diameter	Wall thickness
mean [mm]	1.260	0.163
Std. dev. [mm]	0.131	0.038
N	32	34
min. value [mm]	1.00	0.100
Max. value[mm]	1.55	0.250
*V*	0.104	0.233

**Remarks:** The species described here is different from *Syringopora kowalensis* sp. nov.. It differs by less developed axial canal and visibly smaller corallite diameters. The new species has smaller corallite diameters than most of representatives of the genus. It resembles the most *Syringopora conferta* (Keyserling, 1846) from the Early Carboniferous of the northern Ural Mts. Similar paremetrs are: corallite diameters (1.0–1.5 mm in *S*. *conferta*), corallite spacing (0.3–1.5 mm in *S*. *conferta*) and wall thickness (0.14–0.30 mm in *S*. *conferta*). The latter species has got, however, well developed syrinx, in most cases placed adaxially and in contact with the corallite wall. Besides tabulae in *S*. *conferta* are frequently dissepimental, incomplete. Besides the Early Carboniferous of Belgium, Ural Mountains and Donetsk basin this species was possibly also recorded in the Latest Famennian ("Strunian") of Novaya Zemlya [51], but no further details of the latter locality are given, nor the biometrical data for this Late Famennian material. *Syringopora formosa* Tchudinova, 1974 from the Frasnian of Alaska is very similar by biometric characters (corallite diameter, wall thickness), but the alaskan species has numerous spines arranged in vertical rows [52].

The intracolonial variation of corallite diameters in this species resembles that of the *Syringopora* species described above and heliolitids: on the other hand the wall thickness is somewhat more variable than in the *Syringopora kowalensis* sp. nov.

Order: Auloporida

Family Aulocystidae Sokolov, 1950

Genus: *Aulocystis* Schlüter, 1885

*Aulocystis* sp.

Fig 14

**Fig 14:**
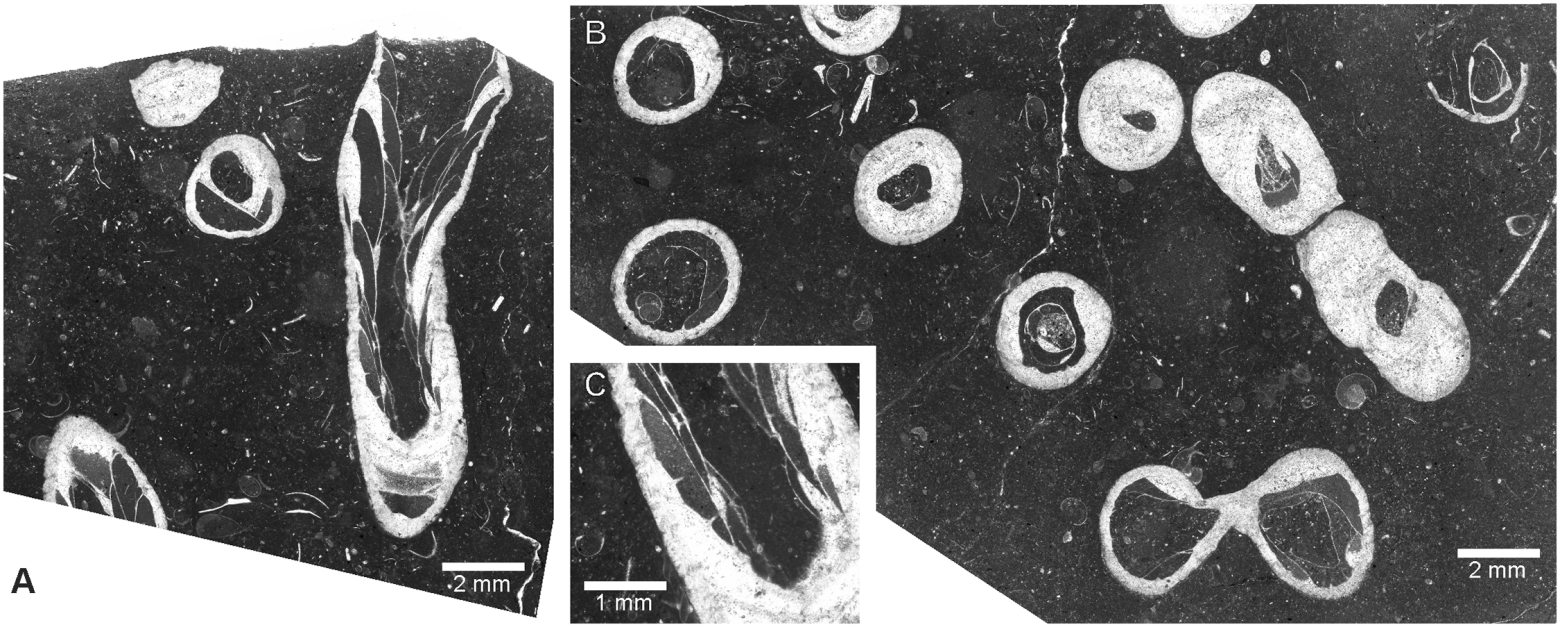
*Aulocystis* sp. from Kowala Quarry, lithological set L, Upper Famennian. (A) Longitudinal section. (B) Detail of the longitudinal section, specimen shown on (A), notice septal and tabular spines. (C) Transverse section. Specimen GIUS 3619 KF 007.

**Material:** One fragment of corallum (GIUS 3619 KF 007), Kowala Quarry, lithological set L, early *Palmatolepis expansa* to *Siphonodella praesulcata* Zones, Upper Famennian, two thin sections.

**Description:** Corallites bent and irregular. They are round or slightly oval in cross section. Calyces deep, twice as deep as wide. Tabulae mostly incomplete, strongly bent, sometimes forming syrinx, which may be axial or adaxial. In cases where corallites are horizontal the tabulae are dissepimental and occur only in the lower part of the corallite, and the lumen of the corallite lies excentrically placed in the upper part of the corallite. Corallite walls uneven, both internal and external surface may be irregularly wrinkled. Connecting tubes absent (only offset connections present). Small, thin and sharp septal spines distributed irregularly; somewhat longer spines occur on tabulae.

**Remarks:** The colony arrangement, lack of connecting elements between corallites of the same generation, axial canal and spines also on tabulae allow generic assignment to *Aulocystis*, but the fragment is too small to be determined to specific level.

## Palaeoecology of Famennian Tabulates from Kowala

The palaeobathymetry of the Famennian Kowala basin was controlled by two processes: global eustatic changes [53], and local synsedimentary block tectonics [22]. The environmental evolution started from possibly shallow Frasnian reefal environments, with photosymbiotic tabulate corals [54], followed by general deepening of the basin, with, however, fluctuations, especially near the Devonian/Carboniferous boundary [22]. The Kowala succesion ends with Early Carboniferous radiolaria-bearing shales representing deep water settings, possibly even thousands of meters deep [55]. The Famennian deposits, at least in their lower part are considered as placed below the photic zone, and consist of dark, clayey and marly deposits [36, 56] with an open marine fauna, such as ammonoids [57], or smooth-shelled rhynchonellid brachiopods commonly regarded as inhabiting deeper water environments [36]. One of the specimens (favositid, a juvenile corallum not determined to generic level) encrusts an orthocone nautiloid (Fig 4), which may provide evidence of its deep water life environment, especially because in these beds cephalopods are common and often well preserved, thus indicating very short transport or no transport et all.

The deep-water character of late Famennian environments can be also emphasized by Thuringian-ecotype ostracods occurring in the *Wocklumeria* limestone of lithological set L. Kozur estimates the upper depth limit for this group as 200–500 m [58]: on the other hand Thuringian-ecotype ostracods from the topmost Famennian of Kowala were probably preferred shallower environments ([40] p. 304–305).

The presence of blind trilobites [27, 37, 59] in complexes J and K may suggest that the seafloor was somewhere about the limit between euphotic and disphotic zone [60]. On the other hand, phacopids with eyes start to appear in the L complex; this may suggest euphotic environment. The opinion presented by Radwański et al. [37] that these late Famennian environments are extremely shallow or even subaerial seems to be isolated. The "desiccation cracks", shown as evidence for such environmental interpretation ([37] text-fig. 7A) may be synaeretic, and microflaser sedimentation may also occur in deeper settings, as was shown for Early Cretaceous pelagic sediments from the Blake-Bahama Basin [61].

The evidence from biomarkers suggests that beds of the uppermost part of the Famennian were deposited in slightly shallower environments. Aryl isoprenoids originating from isorenieratane may indicate the presence of green sulfur bacteria that are typical of photic zone anoxia [56, 62], but they usually occur at the lowermost part of the euphotic zone. The bacteria may also occur in upper parts of the water column as picoplancton. The sampling by Marynowski et al. [56], nevertheless, was not performed in exactly the same beds that yielded the tabulate corals discussed here.

To conclude, it is difficult to assess the bathymetry of late Famennian Kowala settings (especially set L), but most probably these environments were somewhere near or above the limit of the euphotic/disphotic zones.

Specimens of the analyzed corals are well preserved. Especially the edges of calyces are sharp and with preserved fine elements, so these corals are most probably found *in situ* and were not transported from elsewhere.

The intracolonial variation in three species, *M*. *vinni* sp. nov., *S*. *kowalensis* sp. nov. and *S*. *hilarowiczi* sp. nov. is very low, with *V* values of the corallite diameter close to 0.1. Alveolitids occurring in the biohermal and biostromal complexes of the Kowala railroad cut have corresponding values close to 0.2. As intracolonial variation is at least partly controlled environmentally, it may be inferred that corals described here were occurring in rather stable, deeper environments.

The analyzed beds contain crinoid remains. Two coralla of favositids encrust crinoids. At least in one specimen we have obtained a longitudinal section through the pluricolumnal (see Fig 3G). The skeleton adheres to the crinoid skeleton, thus suggesting *syn vivo* encrustation, as in specimens described from the Lower Devonian of Morocco [63].

## Affinities of Faunas

### Relations between Frasnian and Famennian tabulates from Kowala

The Frasnian (below *punctata* Zone, see [3]) tabulate corals from Kowala railroad cut and quarry belong to 18 species. This assemblage is dominated by representatives of Favositida. Pachyporidae are represented by seven species of genera *Thamnopora* and *Striatopora*, Alveolitidae by six species of genera *Alveolites* and *Crassialveolites* and Caliaporidae by possibly single species of ?*Scoliopora* [3]. It must be stressed that *Thamnopora* ex gr. *boloniensis* described from the section may represent several species [3]. The emaining five species are auloporids (Auloporidae: *Aulopora* and Aulocystidae: ?*Adetopora*, [3]). All these faunas occur abundantly in biohermal and biostromal environments. Famennian tabulates described herein are strikingly different. Most papers (e. g. [3, 4, 5]) suggest overall high biodiversity of Frasnian tabulates. The case of Kowala secrion is unusual: on family level Frasnian tabulates are represented by five families, as compared to eight in the Famennian, and only three families are in common. On generic levels in both Frasnian and Famennian eight genera are present for each stage, but it must be stressed that none is common. On specific level the Frasnian biodiversity is nearly twice as large as the Famennian. The comparison of Frasnian and Famennian tabulate faunas from Kowala is given in the Table 5.

**Table 5:** Distribution of tabulate faunas in the Devonian of Kowala Section.

Frasnian		Famennian	
Family	Genera	Family	Genera
**Alveolitidae**	*Alveolites*	**Alveolitidae**	indet.
	*Crassialveolites*		
**Pachyporidae**	*Striatopora*	**Pachyporidae**	*Thamnoptychia*
	?*Striatopora*		
	*Thamnopora*		
**Aulocystidae**	?*Adetopora*	**Aulocystidae**	*Aulocytis*
**Caliaporidae**	*Scoliopora*		
**Auloporidae**	*Aulopora*		
		**Favositidae**	?*Favosites*
		**Cleistoporidae**	?*Yavorskia*
		**Micheliniidae**	?*Michelinia*
		**Syringoporidae**	*Syringopora*
		**Paleacidae**	*Actinotheca*

Data for the Frasnian after [3]. Length of stages not to scale.

Beds near the Frasnian/Famennian boundary at Kowala represent possibly anoxic zones [56]. It is therefore unlikely that faunas present in this area during the Frasnian would continue their presence in such unfavourable conditions. The first Famennian tabulates from the Kowala Quarry, from the set J/K are represented by a single species, *Thamnoptychia mistiaeni* sp. nov., while specimens from the set L represent much more diversified biota. As there is no direct evolutionary link between Frasnian and Famennian tabulates from Kowala it can be assumed that the faunas discussed here more likely represent new recruits than direct descendants of the Frasnian taxa. It can be inferred that few taxa could survive the F/F event in refuges somewhere outside of the modern Holy Cross Mountains, as other Famennian sites in the Holy Cross Mts. are in most cases imply deep environments with ammonoids [33, 34], where the only corals are the sporadically occurring heterocoral *Oligophylloides*. Coral larvae colonized the seafloor of the Kowala basin as soon as conditions became more aerated. Colonization probably took place in two episodes, first with the *Thamnoptychia* assemblage, and later with *Favosites-Syringopora-Michelinia* assemblage.

Kowala quarry and Railroad Cut represent the most complete Late Devonian sequence in the Holy Cross Mountains. It is difficult to explain why the two sites described in the present paper contain such a high biodiversity of tabulate corals, but one of potential reasons is diversified seflor relief and active tectonic processes [31], leading possibly to creation of various niches, where these corals could survive unfavourable conditions. Genera such as *Michelinia*, and *Syringopora* occur both in the pre-Famennian Devonian and the Carboniferous. *Aulocystis*, *Thamnoptychia* and *Favosites* are Devonian; *Yavorskia* is mostly Carboniferous. The Famennian tabulate assemblage from Kowala at the generic level is similar to both older and younger faunas, and links pre- and post-extinction faunas. This might suggest an ancestry for some Carboniferous taxa (michelinids, cleistoporids and syringoporids which are common in the Carboniferous).

The scarcity of tabulate corals in the lower Famennian of is certainly an effect of the F/F event. Subsequent recovery of these fauna, which can be traced in the late Famennian of the Holy Cross Mountains seems to be relatively dynamic. Although, the material of studied tabulate corals is poor in terms of number of specimens, the biodiversity is quite high, even on the higher (generic and family) taxonomic levels. Such a phenomenon was also reported for the Famennian rugose corals, especially the colonial and dissepimented taxa [10–12, 64].

### Relation of Famennian Kowala fauna to "Lower Carboniferous" tabulates from Dalnia

The small, inactive quarries at Dalnia (some 10 km NE from Kowala) display Famennian to Tournaisian neptunian dykes [65]. The detailed study of conodonts from these dykes shown that their age is Upper *C*. *quadrantinodosa* Conodont Zone two dykes): the other dykes span the Middle *costatus* to Carboniferous *crenulata* interval [65]. The dykes yielded rich fauna of conodonts, trilobites, rugose and tabulate corals. The latter ones were described by Stasińska [66] who identified four species (all of them new): *Emmonsia dalniae* Stasińska, 1973; *Michelinopora szulczewskii* Stasińska, 1973; *Acaciapora infracarbonica* Stasińska, 1973 and *Kueichowpora polonica* Stasińska, 1973. Only *A*. *infracarbonica* was found together with conodonts indicating Tournaisian age; others were found in the rubble and were devoid of sediment. Their age was estimated as not older than *costatus* Zone ([65] p. 25), now middle *expansa* to middle part of *praesulcata* Zones. Nevertheless, Stasińska ([66] p. 83) gave a “Lower Carboniferous” age for the whole tabulate fauna, but in the indication of a *stratum typicum* for each new species she suggested a broader stratigraphic range: "*Wocklumeria* or *Gattendorfia* stage" ([66] p. 84–87).

As the age of material described by Stasińska [66] is not precisely determined and some of the taxa might also represent late Famennian faunas, the Famennian tabulate faunas of the Southern Region of the Holy Cross Mountains may even be richer than concluded in this paper.

Faunas described by Stasińska [66] are slightly younger than or contemporaneous those described here. As they were described from a site that was somewhat distant (in palinspastic reconstruction more than today's 10 km; strikes of most variscan folds are about 120°), both faunas may be related. The anatomical features of "*Michelinopora" szulczewskii* may suggest a relationship to the *Favosites* described here, but the biometric features of the former species are different (smaller corallite diameters, large mural pores, evenly spaced, flat tabulae). The status of the genus *Michelinopora* is unclear [67] and it could be considered as junior subjective synonym of *Michelinia* [18]. *Acaciapora infracarbonica*, by corallum organization, corallum size, and presence of septal ribs could be considered a descendant of "*Thamnoptychia*" described here. *Emmonsia dalniae*, with squamulae and small diameters certainly has no unequivocal relation to Kowala faunas. *Kueichowpora polonica* was described on the basis of a single, small fragment of corallum and this determination is doubtful, so it is difficult to assess its relationships.

### Relation of the Famennian Kowala fauna to other Famennian tabulate faunas

As stated in the introduction above Famennian faunas are very rare worldwide. The classical late Famennian ("Strunian") faunas from Etroeungt consist of four species [6]: one *Vaughania*, one *Yavorskia* and two *Syringopora* species [14]. Faunas described herein are very similar, however their biodiversity is much higher. A single species of *Cleistopora* is known from the Famennian of Rhenish Slate Mountains [68]. In Poland, apart from the Holy Cross Mts., Famennian tabulates (several specimens of syringoporoids, undescribed) were recorded only in Dzikowiec in the Sudetes [12]. It seems probable that Famennian tabulates from Kowala and Ostrówka form an assemblage with the highest biodiversity recorded so far in the Famennian.

On the generic level, the Famennian fauna from Kowala is very similar to late Famennian to early Tournaisian faunas from Omolon [16, 45]. The latest Famennian of Omolon contains two *Syringopora* species, but in the early Tournaisian such genera as *Yavorskia*, *Michelinia*, *Ortolithes*, *Fuchungopora*, *Roemeria and Thecostegites* appear. The biodiversity is similar, containing (for Famennian and Tournaisian together) 11 species (Smirnova in [45]).

It must be emphasized that Famennian tabulates described in this paper do not appear immediately after the Frasnian/Famennian limit. They are at least 10Ma younger [36]. This may explain why the biodiversity is than elsewhere, as tabulate corals had had sufficient time to recover after the crisis. On the other hand, coeval “Strunian” beds from Etroeungt have yielded fewer species.

## Conclusions

1. The Famennian tabulate fauna from Kowala consists of 10 species (eight described here and two known previously) and represents the richest Famennian collection described so far from the Famennian, after the F/F crisis.
2. One of species, *Thamnoptychia mistiaeni* sp. nov. also occurs in the Famennian of Ostrówka.
3. Faunas described here most probably inhabited deeper water settings, near the limit between euphotic and disphotic zones or slightly above.
4. The tabulate faunas described here are on the generic level intermediate between Devonian and Carboniferous faunas, and this might suggest their ancestry for at least several Carboniferous genera. This is emphasized by similarities to tabulate faunas from the earliest Tournaisian of Omolon (NE Russia).
5. The basin, which developed during the Famennian in the area of today’s Holy Cross Mountains was certainly not a refuge during the F/F crisis for corals, due to a prolonged interval of local anoxia of seawater. Hence, tabulate taxa described here represent new recruits and have no direct evolutionary linkage to Frasnian faunas from the same area.
6. The colonization took place in two episodes: first was a monospecific population of *Thamnoptychia*, adiversified *Favosites-Syringopora-Michelinia* assemblage came later.
